# Supplementary figures and images for: Multiplexed assay of variant effect reveals residues of functional importance in the BRCA1 coiled-coil and serine cluster domains
Source: PLoS One. 2023 Nov 2;18(11):e0293422. doi: 10.1371/journal.pone.0293422 (PMC10621863; doi:10.1371/journal.pone.0293422)

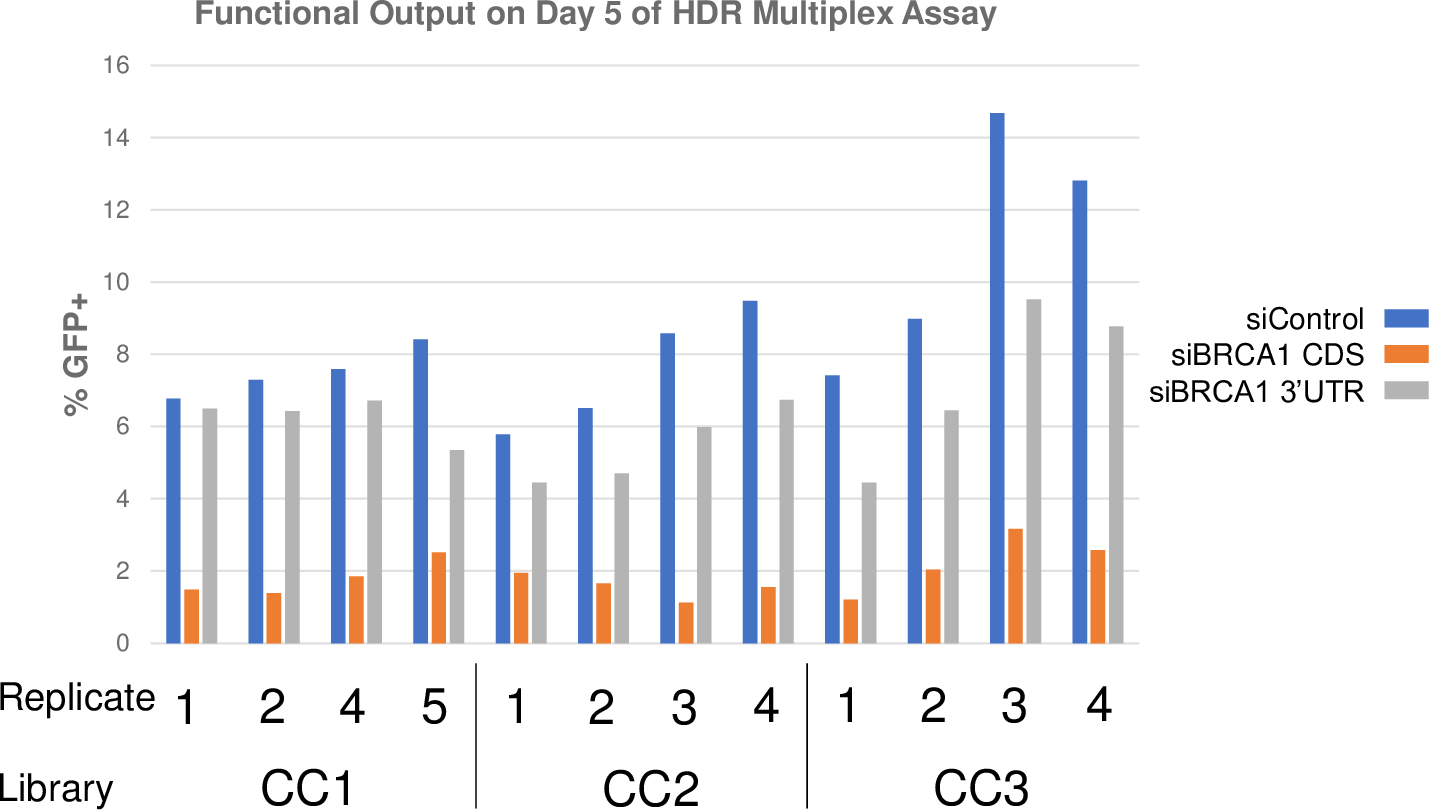

Supplement: S1 Fig — Before sorting into GFP-positive and negative cohorts and extracting genomic DNA on Day 6, different siRNA conditions were evaluated for GFP-positive percentage on Day 5 of the multiplexed assay. siBRCA1 3’UTR samples scored a lower percentage than siControl, as it is expected that each library will have some loss of function variants. siBRCA1 coding specific (CDS) scored low as both the endogenous BRCA1 and integrated BRCA1 variant were silenced. (TIF) [file pone.0293422.s001.tif]

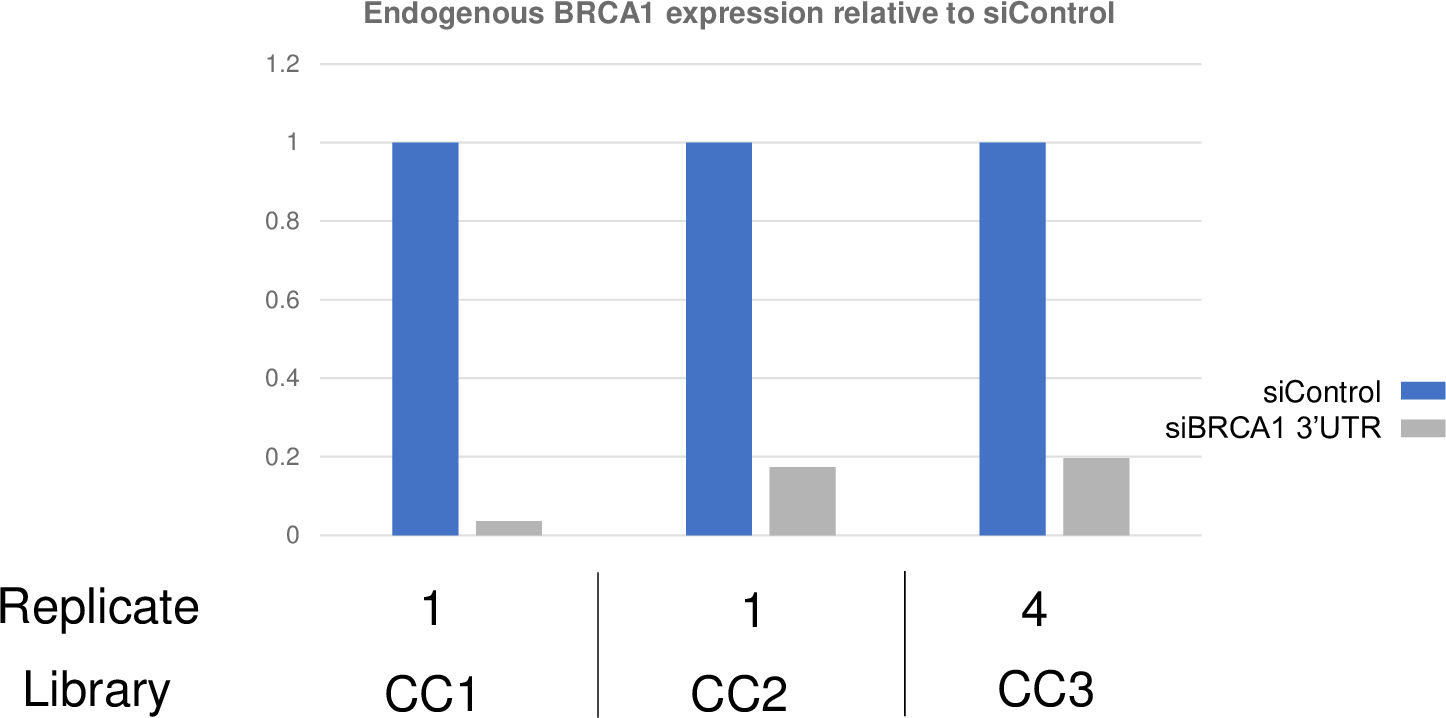

Supplement: S2 Fig — Examples are shown for endogenous BRCA1 expression for siControl and siBRCA1 3’UTR treated samples in each multiplexed replicate. (TIF) [file pone.0293422.s002.tif]

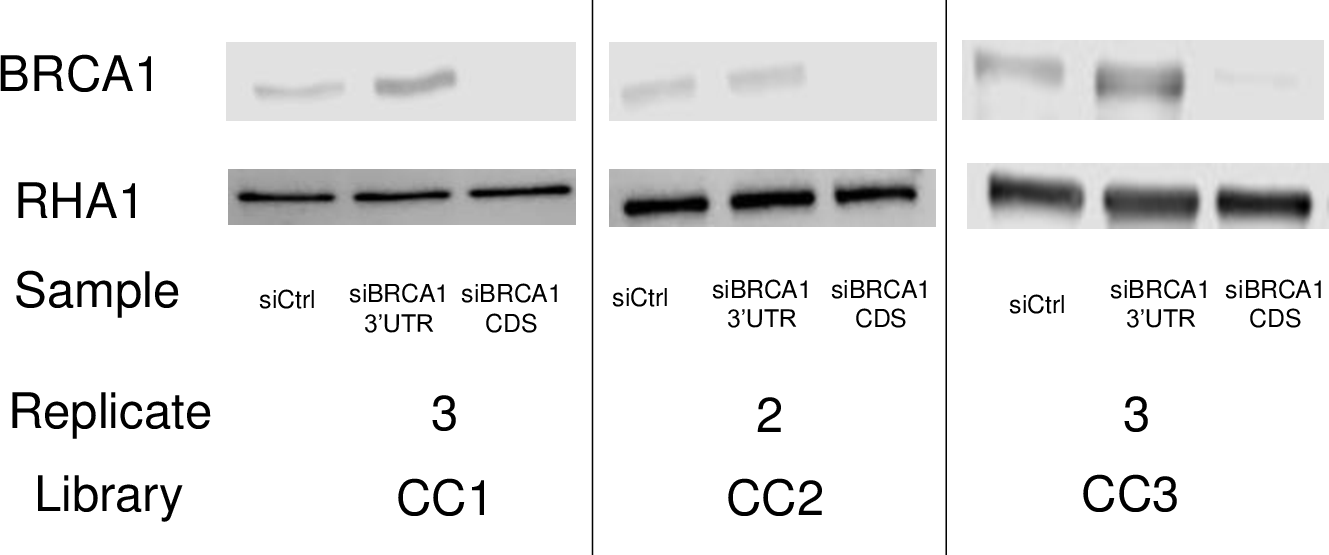

Supplement: S3 Fig — Examples are shown for the analysis of BRCA1 expression for siControl, siBRCA1 3’UTR, and siBRCA1 CDS samples for one replicate per library. RHA1 is an antibody specific for RNA Helicase A and detects a 140 kDa protein used as a loading control. (TIF) [file pone.0293422.s003.tif]

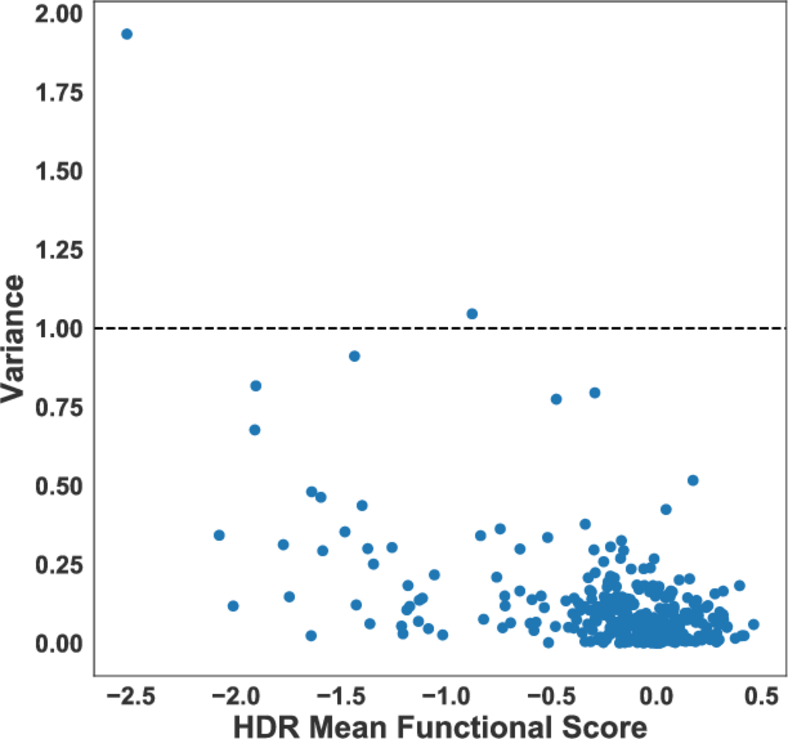

Supplement: S4 Fig — Variants are plotted by their functional score in the HDR assay against variance of the score between replicates. Any variants with a variance greater than 1 were not included in the final dataset. (TIF) [file pone.0293422.s004.tif]

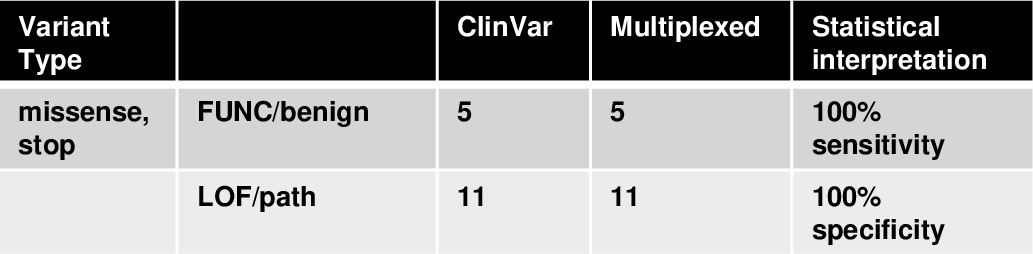

Supplement: S5 Fig — Of the five variants with a benign or likely benign ClinVar designation, all five scored as functionally normal in the multiplexed assay, indicating 100% specificity. Of the 11 variants with a pathogenic or likely pathogenic ClinVar designation, all 11 scored as LOF in the HDR assay, indicating 100% sensitivity. (TIF) [file pone.0293422.s005.tif]

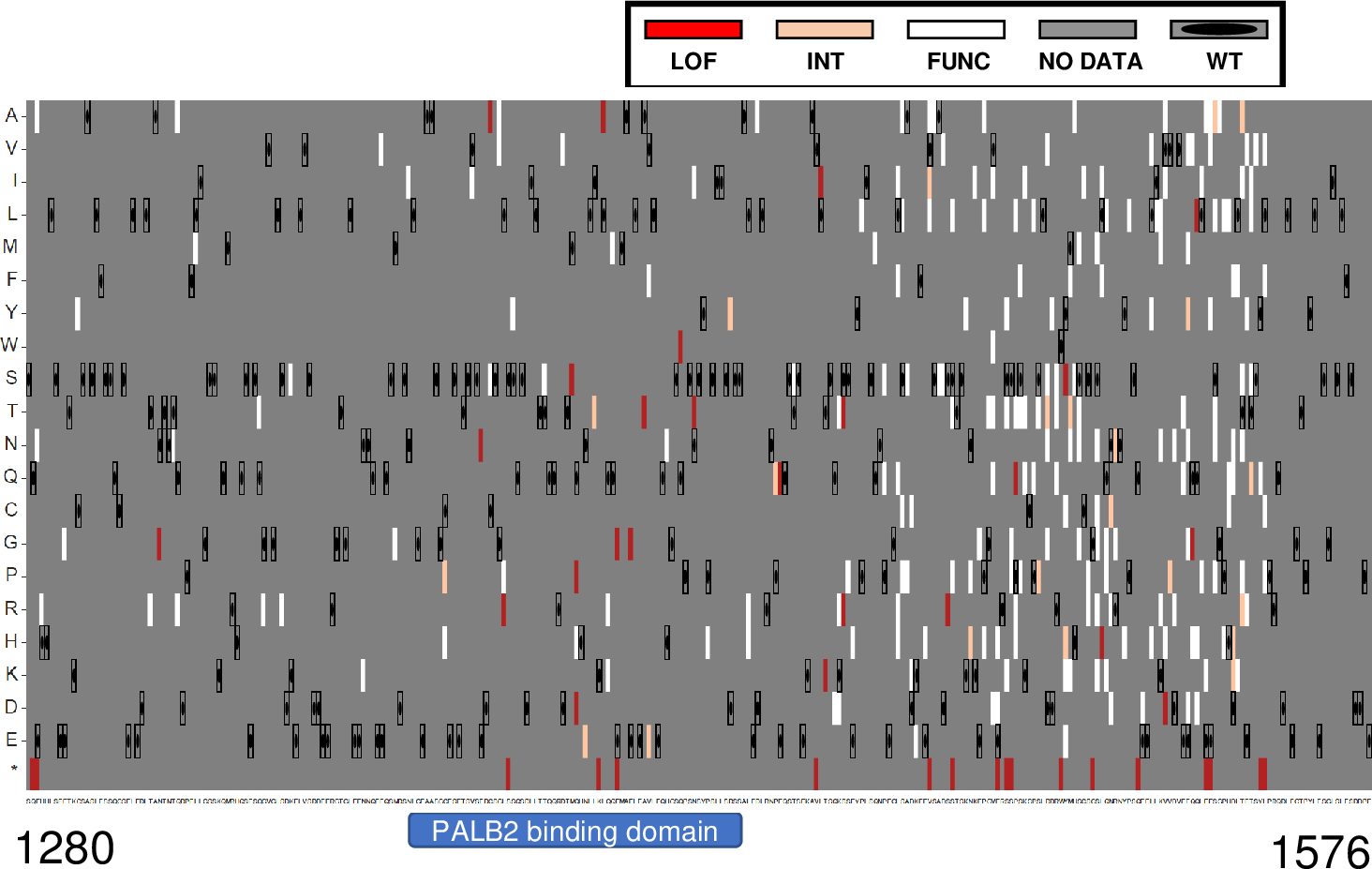

Supplement: S6 Fig — After categorization as LOF, intermediate function, or functionally normal, variants were graphed according to their position on the BRCA1 protein (x-axis) and the substituted amino acid (y-axis). Red indicates LOF, pink indicates intermediate function, and white indicates functionally normal. Black outlines with a black dot indicate WT amino acid, and gray indicates insufficient data to score the function. (TIF) [file pone.0293422.s006.tif]
