# Supplementary material for: Multiplexed assay of variant effect reveals residues of functional importance in the BRCA1 coiled-coil and serine cluster domains
Source: PLoS One. 2023 Nov 2;18(11):e0293422. doi: 10.1371/journal.pone.0293422 (PMC10621863; doi:10.1371/journal.pone.0293422)
Supplement: S1 Raw images — Immunoblots used in S3 Fig are shown for the full membrane. BRCA1 and RHA1 stains for CC1 replicate 3, CC2 replicate 2, CC3 replicate 3, and CC3 replicate 4. (PDF) [file pone.0293422.s007.pdf]

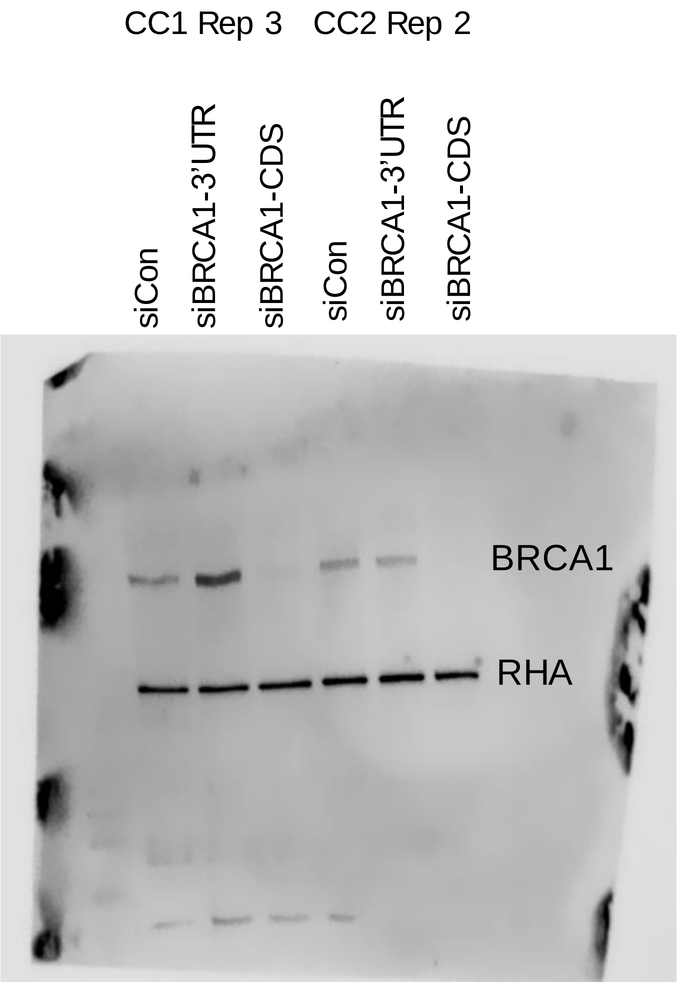
CC1 Rep 3
CC2 Rep 2
siCon
siBRCA1-3'UTR
siBRCA1-CDS
siCon
siBRCA1-3'UTR
siBRCA1-CDS
BRCA1
RHA

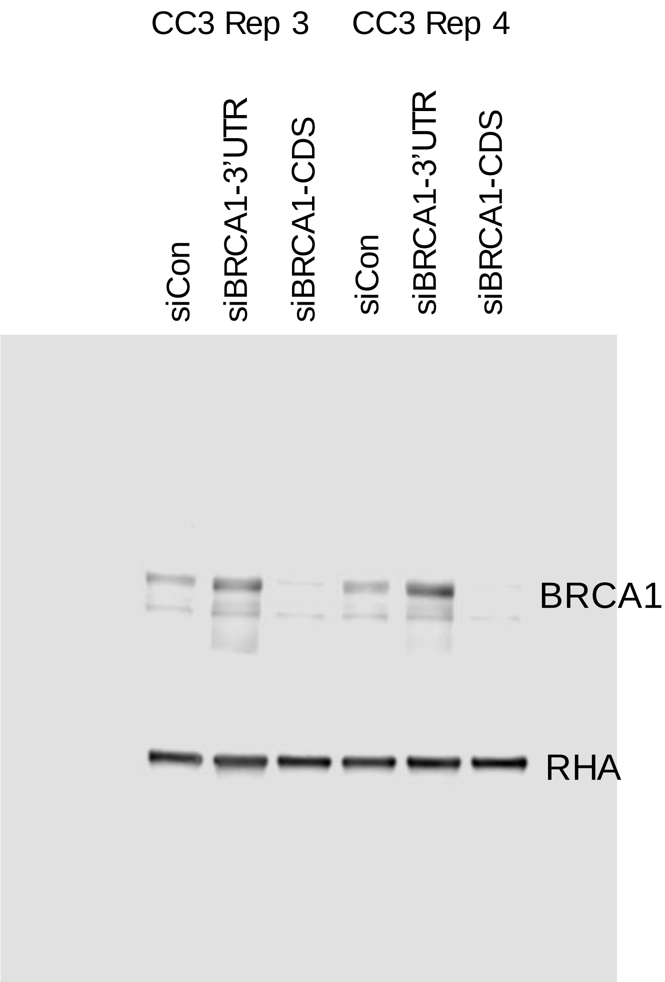
CC3 Rep 3
CC3 Rep 4
siCon
siBRCA1-3'UTR
siBRCA1-CDS
siCon
siBRCA1-3'UTR
siBRCA1-CDS
BRCA1
RHA
