# Supplementary material for: Multiplexed assay of variant effect reveals residues of functional importance in the BRCA1 coiled-coil and serine cluster domains
Source: PLoS One. 2023 Nov 2;18(11):e0293422. doi: 10.1371/journal.pone.0293422 (PMC10621863; doi:10.1371/journal.pone.0293422)
Supplement: Attachment — Submitted filename: Response to Reviewers BRCA1CC 2023 September 5.docx [file pone.0293422.s009.docx]

Dear reviewer,

Thank you for your careful consideration and thoughtful comments of our manuscript. We have included in this letter our changes made to the manuscript since first submission. We believe that your suggestions and the subsequent changes made have improved the quality of the paper. Please see below for specific changes enacted for this resubmission.

We have attached a Word file for the compare documents, and we wish to point out that it looks like whole blocks of text have changed, and this is likely due to enough small edits on the reference numbers to cause entire paragraphs to be indicated as new. We apologize that this effect diminishes the usefulness of the tracked changes on the document comparison.

2. In your Methods section, please report the source of HeLa cells used for your study.

We have now stated this in the Methods section (line 118-119).

6. In your cover letter, please note whether your blot/gel image data are in Supporting Information or posted at a public data repository, provide the repository URL if relevant, and provide specific details as to which raw blot/gel images, if any, are not available. Email us at [plosone@plos.org](mailto:plosone@plos.org) if you have any questions.

We have included the full blots as figure S7.

1. The authors should provide a simple table listing 1) each variant tested (310?), 2) whether it was tested by the multiplexed and/or singlet assay, 3) the observed HDR activity (i.e., function, intermediate, nonfunctional), and 4) whether it is currently listed in the ClinVar database. It may be possible to ferret out all this information by working through the current tables, but it would be a rather painful exercise.

We have included the requested table as Table S2. The final dataset consisted of 310 variants tested in the multiplexed assay, of which 5 had singleton scores performed by the authors and 1 had a singleton score from a 2015 Parvin lab paper (cited in-text). The final dataset also included 20 variants tested in the singleton assay but not included in the final multiplexed dataset for a total of 330 variants evaluated.

2. This segment of BRCA1 includes the Palb2-binding sequences, and the authors’ data nicely underscore the important of this interaction in HDR and tumor suppression. However, this segment also encompasses the poorly-understood, but intriguing, cluster of SQ/TQ phosphorylation sites recognized by the ATR/ATM kinases. Interestingly, on lines 319-321, the authors mention that five of the nonfunctional missense variants affect either serine, threonine, or tyrosine. However, they don’t identify them. Given their potential significance, the specific variants should be identified in the text and noted as to whether they correspond to SQ/TQ sites. And if so, then some discussion of the literature would be warranted, such as whether the site is known to be phosphorylated by ATR/ATM and whether phosphorylation has functional consequences.

We have clarified and explicitly listed the five nonfunctional missense variants at a Ser/Thr/Tyr outside of the PALB2-binding domain and have stated that none of them appear as a part of an SQ/TQ motif. Quick discussion is given to the fact that two residues, S1460 and S1483, have been documented as phosphorylated, but that the functional consequence of this is not clear. (lines 326-333)

3. Is Figure 5 necessary? At least for me, it doesn’t provide any information.

This figure is now figure S6.

4. Do the authors wish to mention “Figure S4” in the sentence on lines 223-226?

We now mention Figure S4 at the end of the section titled “Determination of BRCA1 variant function in DNA repair from assays score”. (lines 246-247)

5. On lines 347-350, the authors state that “The ClinVar database lists pathogenic or 348 likely pathogenic missense variants in this 297-codon region that affect splicing at residue 1495 and residue 1559”. Is there a reference describing these splicing results?

We now include a reference indicating differential splicing due to the BRCA1-p.R1495M variant, and state that due to the location of the other variants at residues 1495 and 1559 at the exon-intron boundary, it can be inferred that differential splicing also occurs with these variants. (lines 359-367)

Sincerely,

Gregory Nagy et al.
