# Supplementary material for: Multiplexed assay of variant effect reveals residues of functional importance in the BRCA1 coiled-coil and serine cluster domains
Source: PLoS One. 2023 Nov 2;18(11):e0293422. doi: 10.1371/journal.pone.0293422 (PMC10621863; doi:10.1371/journal.pone.0293422)
Supplement: Attachment — Submitted filename: PLOS ONE Response to Reviewers 2023 October 1.docx [file pone.0293422.s010.docx]

Dear reviewer,

Thank you for your careful consideration and thoughtful comments of our manuscript. We have included in this letter our changes made to the manuscript since our first resubmission. We believe that your suggestions and the subsequent changes made have improved the quality of the paper. Please see below for specific changes enacted for this second resubmission.

1.The authors do not mention how functional analyses of missense variants are used in the variant interpretation guidelines following the original ACMG-AMP guidelines (Richards, 2015. PMID: 25741868), or by the more newly released BRCA1 specific guidelines published by CanVIG-UK ([https://www.cangene-canvaruk.org/_files/ugd/ed948a_f05b6f7767ad499a8539131d06035432.pdf](https://urldefense.com/v3/__https:/www.cangene-canvaruk.org/_files/ugd/ed948a_f05b6f7767ad499a8539131d06035432.pdf__;!!AU3bcTlGKuA!Gl4CDhSGg6_n7bUT2P8YjWCmL_a_lEKXU9TLsx1WJyonzzZ_HqUPUUh4DKUylyaiIiV07h_qAiQDwQYknE8ipQQ$)) or ClinGene/Enigma ([https://cspec.genome.network/cspec/ui/svi/doc/GN092](https://urldefense.com/v3/__https:/cspec.genome.network/cspec/ui/svi/doc/GN092__;!!AU3bcTlGKuA!Gl4CDhSGg6_n7bUT2P8YjWCmL_a_lEKXU9TLsx1WJyonzzZ_HqUPUUh4DKUylyaiIiV07h_qAiQDwQYkMzfFWrk$)). This topic should be introduced in the introduction.

We now discuss the updated guidelines in a new paragraph in the introduction (lines 81–97) and included the references listed above.

2. Line 74: In this 297-residue region there are greater than 350 missense VUS in ClinVar. Change to: In this 297-residue region there are more than 350 missense VUS in ClinVar.

We have made this change (line 74).

3. Line 222-226: Cells were sorted based on GFP fluorescence, and genomic DNA was subsequently extracted to identify the integrated BRCA1 variant in each cell. The frequencies of variants in the GFP-positive and input cohorts were determined and a functional score for each variant relative to WT was calculated based on variant distribution between the GFP-positive and input cohorts using Enrich2 software (20). This paragraph belongs to the Method section in my opinion.

We have moved this text to the Methods section (lines 158-160 and 164-166).

4. Line 231: The synonymous variants they used to define “normal” thresholds, are they listed somewhere? If not, please do so. Did the authors check that these variants do not affect splicing or do not involve conserved nucleotides?

To clarify, the cutoffs themselves were defined not by synonymous variants, but rather by the missense variants in the control siRNA condition, as well as the nonsense variants in the endogenous *BRCA1*-siRNA condition. The synonymous variants were indeed used as a control in that they were evaluated to ensure a maintenance of normal distribution around a score of zero for synonymous variants in the endogenous *BRCA1* siRNA condition. To the reviewer’s comment, we have added Table S10 (lines 251 and 268-270) which includes the nucleotide changes of the synonymous variant and corresponding assay scores. Due to the fact that these synonymous variants are being expressed in a *BRCA1* cDNA construct in a genomically recombined plasmid and not at the *BRCA1* endogenous locus, there is no need to check for splicing effects of the synonymous variants.

5. The authors used ClinVar classifications to compare the functional multiplexed results, but did the authors include all Clin Var classifications regardless of i) year of classification, ii) amount of ClinVar stars, iii) where any ClinVar classifications by expert panels? It would be nice if this information could be included in Suppl Table 2 and also in the Methods section. The same question goes for Figure 4 (conflicting interpretations).

We have added Table S11 (lines 338-340) which includes details of the ClinVar entry of a variant, including year of last evaluation, number of stars in the ClinVar designation, and whether the ClinVar designation was reviewed by an expert panel.

6. Line 310: This percentage (3.0%, 2 of 66) of VUS and conflicting that scored as loss of function was lower than the corresponding percentage (5.8%, 14 of 240) of the RING domain and amino-terminal evaluation (12). The authors need to specify what they mean by conflicting? Conflicting between which categories, VUS vs benign, VUS vs likely benign, VUS vs likely pathogenic, etc?

We have clarified (lines 336-338) that the variants having a Conflicting interpretation on ClinVar have entries as Uncertain Significance, Likely Benign, or Benign. The number of classifications of each category for a variant entry are included in Table S11.

7. Figure 3: Could they mark their cutoff thresholds (0.4 and 0.7) as lines in the figure?

We have made this change.

8. Suppl table S2: This is a very useful table and will be used by clinical/laboratory geneticist working with variant interpretation of rare BRCA1 variants. Could the authors include which reference transcript for BRCA1 they are referring to and also include the nomenclature at the DNA level?(1)
For instance: Q1281* should be written as BRCA1 (NM_007294.4): c.3841C>T Q1281* or c.3841C>T Gln1281* (Three –letter amino acid codes are preferred by HGVS guidelines (Sequence Variant Nomenclature (hgvs.org)).

We have now stated the reference transcript (line 101) for BRCA1. We made the change of switching amino acid notation from one letter to three letter codes for the manuscript and supplemental information. Regarding the inclusion of the nucleotide-level changes for each variant, we have restricted this to synonymous variants only (lines 341-351). Our rationale is that for missense and nonsense variants, the focus should be on the amino acid change rather than the nucleotide change, given (1) the genomic context of these variants being expressed in a *BRCA1* cDNA construct either as part of a genomically recombined plasmid (multiplexed) or expressed transiently from a cytosolic plasmid (singleton); neither of these is from the endogenous locus of *BRCA1* and (2) the fact that this manuscript evaluates a functional impact of a BRCA1 variant on DNA repair at the protein level. Additionally, while not included in the supplemental tables, the nucleotide-level changes for these variants are available on Gene Expression Omnibus in the long-read sequencing file that details the barcode-variant pairings.

Thank you for your continued consideration of this manuscript for publication in PLOS ONE.

Sincerely,

Gregory Nagy et al.
